# Prevalence and Factors Associated with Intestinal Parasitic Infections among Pregnant Women in West Gojjam Zone, Northwest Ethiopia

**DOI:** 10.1155/2020/8855362

**Published:** 2020-08-06

**Authors:** Tadesse Hailu, Bayeh Abera, Wondemagegn Mulu, Simachew Kassa, Ashenafi Genanew, Arancha Amor

**Affiliations:** ^1^Department of Medical Laboratory Science, College of Medicine and Health Science, Bahir Dar University, P.O. Box: 79, Ethiopia; ^2^Department of Midwifery, College of Medicine and Health Sciences, Bahir Dar University, P.O. Box: 79, Ethiopia; ^3^Department of Pharmacy, College of Medicine and Health Sciences, Bahir Dar University, P.O. Box: 79, Ethiopia; ^4^Mundo Sano Foundation, Institute of Health Carlos III, Spain

## Abstract

**Background:**

Intestinal parasitic infections are the major causes of morbidity and mortality in sub-Saharan countries. The disease burden of these parasites is significantly high among pregnant women in developing countries like Ethiopia. Poor living conditions, sanitation, and hygiene are believed to be the contributing factors. The aim of this study was to determine the magnitude of intestinal parasitic infection and factors associated with pregnant women.

**Methods:**

A cross-sectional study was conducted from February 2017 to June 2017. A structured questionnaire was used to obtain the sociodemographic and other explanatory variables via face-to-face interviews. Stool samples were collected and examined using formol ether concentration technique. The magnitude of parasitic infection was calculated using descriptive statistics. The association between intestinal parasitic infection and determinant factors was assessed by logistic regression. The differences were considered to be statistically significant if the *p* value was less than 0.05.

**Results:**

From a total of 743 pregnant women, the overall prevalence of intestinal parasitosis was 277 (37.3%). The prevalence of hookworm 138 (18.6%) was the leading cause of intestinal parasitosis followed by *E*. *histolytica*/*dispar* 113 (15.2%). Dwelling in rural area (AOR: 2.9 (95% CI: 1.85-4.85)), being a farmer (AOR: 1.91 (95% CI: 1.20-3.03)), eating raw vegetables (AOR: 1.45, 95% CI:0.09-0.24), lack of proper use of latrine (AOR: 2.89 (95%1.18-7.08)), poor environmental sanitation (AOR: 0.19 (95%: CI:0.08-0.47)), habit of soil eating (AOR: 0.42 (95% CI: 0.25-0.72)), having irrigation practice (AOR: 0.47 (95% CI: 0.29-0.77)), and lack of health education (AOR: 0.32 (95% CI: 0.13-0.77)) were significantly associated with intestinal parasitic infections.

**Conclusions:**

Intestinal parasitic infection is a major problem among pregnant women in the study area. High parasitic infection is associated with poor hygienic and sanitation practices. Therefore, awareness creation through health education should be given to pregnancy on intestinal parasitic infection and associated factors.

## 1. Background

Intestinal parasitic infections caused by protozoa and geohelminths are common problems in the human population, especially in resource-poor countries. *Amoebiasis*, *ascariasis*, hookworm infection, and trichuriasis are among the ten most common intestinal parasitic infections in the world [1]. Globally, soil-transmitting helminth (STH) infections are the main intestinal parasitic infections. Approximately, 4.5 billion people are at risk, more than 1 billion people become infected, and 450 million are ill from STHs [2]. High prevalence of STHs is mainly related to poverty, poor living conditions, personal and environmental hygiene, sanitation, and water supply facilities [3].

STH infections are the major causes of morbidity and mortality among pregnant women [4] since infected pregnant women develop malnutrition, maternal anemia, and increased vulnerability to other infections [5, 6]. STH infections during pregnancy may also be associated with adverse outcomes on the offspring including low birth weight, intrauterine fetal growth restriction, and perinatal mortality [6]. STHs, especially hookworm parasite, cause total energy, protein, folate, and zinc loss in pregnant women [7]. As a result, low pregnancy weight gain and intrauterine fetal growth restriction, followed by low birth weight and higher perinatal mortality rates happened in pregnant women [8].

Lack of proper sanitation and hygiene, the habit of eating raw vegetables, walking barefoot, and water source are some of the factors associated with the STH infections [9]. The impact of STH infections will sustain in the environment if we cannot address factors associated with STH infections. So, prevention of STHs is possible through health education on effective sanitation and hygiene of STHs since the benefit of deworming during pregnancy has not been rigorously evaluated. The effect of anthelminthic drug use during pregnancy on maternal anemia, birth weight, perinatal mortality, or congenital anomalies was low [10].

Institution-based information revealed that infection with STHs is the primary disease among pregnant women in the study area but the prevalence and factors associated with STH infections are still unknown in the West Gojjam Zone. To minimize the impact of STH infections during pregnancy in the study area, determining the magnitude of STH infections with their associated factors should be prioritized. This helps to give evidence-based propositions for timely interventions.

## 2. Material and Methods

### 2.1. Study Design, Period, and Area

A cross-sectional study was conducted from February 2017 to June 2017 among pregnant women in West Gojjam Zone, Amhara Regional State, Northwest Ethiopia. The average elevation of the zone is 2,300 meters. The annual temperature of the study area ranges from 16.68°C to 37.6°C. The samples were collected from five woredas of West Gojjam Zone, namely, Bure, Debub Achefer, Finote Selam, Jabi Tehnan, and Mecha woredas. The respective health centers in which we collected data were Bure, Durbete, Finote Selam, Mankusa, and Wotet Abay. A total of 743 pregnant women were included in this study. The sample size in each woreda was proportionally allocated.

The five woredas of the West Gojjam Zone selected considering urban and rural settings. One health center was selected from each woreda based on their laboratory facilities and the client flow they have. The calculated sample size allocated to each health institution was according to their population size in the catchment areas. The random sampling technique was used to include 743 study participants.

### 2.2. Study Population

All pregnant women attending the antenatal clinic for the first time visit and living in West Gojjam Zone were randomly selected and included; however, pregnant women undertaking antihelminthic drugs during the time of data collection were excluded from the study.

### 2.3. Data Collection

Data on sociodemographic variables (age, residence, occupation, and religion) and environmental-related factors (sanitation practice, latrine usage, soil eating habit, walking barefoot, and eating raw vegetables and irrigation) were collected by face-to-face interviews using a structured questionnaire. The data was collected by trained midwifery health professionals.

### 2.4. Laboratory Data Collection and Examination

Freshly passed stool specimens were collected it among pregnant women in the health institutions using clean plastic cups at the health institutions. The stool cups were labeled by serial card number. The laboratory professionals took part in all processes of stool collection and examination. The stool samples were processed for microscopic examination using formol ether concentration techniques (FECT). The stool examination was done in the health institution laboratory.

In FECT, the stool sample (0.5 g) was transferred into 10 ml of normal saline in a glass container and mix thoroughly. Two layers of gauze were placed in a funnel and strained the contents into a 15 ml centrifuge tube. Then, 2.5 ml of 10% formaldehyde and 1 ml of ether were added to a test tube. The test tubes were mixed well and centrifuged at 1,000 revolutions for three minutes. The supernatant was removed and the sediment was mixed well, prepared on two slides one with saline and the other with iodine, and covered with cover slide and detected under a microscope.

### 2.5. Data Quality Assurance

To ensure reliable data collection, training on data collection and examination as well as explanation about the study were given before sample collection. Filled questionnaires were collected after checking for consistency and completeness. Application of standard procedures during the data collection process and accuracy of test results were supervised by the principal investigator. Specimens were cross-checked by principal investigators to increase the accuracy of laboratory results. To eliminate observer bias, stool slides were examined independently with two experienced laboratory professionals and 10% of the FECT slides were randomly selected and read by other laboratory professionals as quality control.

### 2.6. Data Analysis

Data were entered and analyzed using Statistical Package for Social Science (SPSS) version 22. The overall magnitude of geohelminthic infection was analyzed using descriptive statistics of the sample through frequencies and cross-tabulations. Associations between dependent and independent variables were analyzed by binary logistic regression. Variables with *p* < 0.2 in the binary regression were selected and analyzed by multivariable analysis using a backward elimination method to avoid the cofounding effect and calculating the odds ratios (OR) at 95% confidence intervals (CI). In all statistical tests, the differences were considered to be statistically significant if the *p* value was less than 0.05.

### 2.7. Ethical Consideration

The proposal was ethically approved by the Ethical Review Committee of Bahir Dar University, College of Medicine and Health Science. Permission letters were obtained from the Amhara Regional Health Bureau, West Gojjam Zonal health office and provided to the specific study area to conduct the research. Written informed consent was obtained from every study participant. Participants who tested positive for any parasitic infections got appropriate treatment accordingly from the responsible body.

## 3. Results

### 3.1. Sociodemographic Characteristics of the Study Subjects

A total of 743 pregnant mothers were interviewed with a response rate of 100%. The median age of mothers was 25 years (range: 15-45). Most (466 (62.7%)) study participants were in the age ≥ 35 years. The majority (62.3%) of pregnant women were rural dwellers (Table 1).

### 3.2. Magnitude of Intestinal Parasitosis

The overall prevalence of intestinal parasitosis among pregnant women was 277 (37.3%). The prevalence of intestinal parasitosis among pregnant women in Bure, Debub Achefer, FinoteSelam, Jabi Tehnan, and Mecha woredas was 44 (5.9%), 48 (6.5%), 85 (11.4%), 43 (5.8%), and 56 (7.7%), respectively. Hookworm was the highest prevalent 138 (49.8%) among the parasitic-infected pregnant women followed by *E*. *histolytica/dispar* 113 (40.8%) and *G*. *lamblia* 53 (19.1%) in West Gojjam (Table 2).

The prevalence of double infection among pregnant women was 31 (11.2%). The highest prevalence of double infection was found in hookworm and *E*. *histolytica* 12 (4.3%) followed by *E*. *histolytica/dispar* and *G*. *lamblia* 11 (3.9%). Triple infection with hookworm, *G*. *lamblia*, and *E*. *histolytica/dispar* was found in only 3 individuals (Table 2). The prevalence of intestinal parasitic infection among rural and urban dwellers was 25.2% and 12.1%, respectively (Table 2).

### 3.3. Associated Factors of Intestinal Parasitic Infection

In terms of multivariate analysis, intestinal parasitic infections were significantly associated with living in rural area (AOR: 2.99 (95% CI: 1.85-4.85)), being a farmer (AOR: 1.91 (95% CI: 1.20-3.03)), eating raw vegetables (AOR: 1.45 (95% CI: 0.09-0.24)), and lack of proper use of latrine (AOR: 2.89 (95% CI: 1.18-7.08)). The odds of intestinal parasitic infection were 81% lower in pregnant women who keep environmental sanitation than pregnant women that did not clean their environment (AOR: 0.19 (95% CI: 0.08-0.47)). Pregnant women who did not have the habit of eating soil were 58% protected from intestinal parasites infection than those who had the habit of eating soil (AOR: 0.42 (95%CT: 0.25-0.72)). The odds ratio of intestinal parasitic infection was 53% lower in pregnant women who did not have irrigation practice than those who had irrigation practice (AOR: 0.47 (95% CI: 0.29-0.77)). Pregnant women who got health education were 68% protected from intestinal parasitic infection than those who did not get health education about intestinal parasite (AOR: 0.32 (95% CI: 0.13-0.77)) (Table 3).

## 4. Discussion

Intestinal parasites are important disease-causing agents in pregnant women. Their impacts rest upon not only on the health of pregnant women but also on her offspring. The most important factors that determine the prevalence of intestinal parasitic infections are associated with hygiene and sanitation [11].

The overall prevalence of intestinal parasitic infection among pregnant women was 37.3% in the present study. This result was higher than studies done in Gondar town, Ethiopia [12] and Bahir Dar city, Ethiopia [13], but lower than a study conducted in Addis Ababa [14]. The difference might be due to differences in sanitation and hygiene practice in different parts of the country. Also, most participants of the present study were from a rural area where low educational status and poor sanitation and hygiene were commonly practiced.

The prevalence of intestinal helminthic infection among pregnant women was 19.5% in the present study. This result was comparable with previous studies done in Kenya [15] and South Gondar, Ethiopia [16]. However, it was lower than a study done in East Wolega, Ethiopia [17]. The difference might be due to the differences in the distribution of helminths from place to place or from one geographical area to another. The temperature, soil type, rainfall, altitude, and humidity are also the major environmental factors that influence the preexistence of helminthic infections in one geographical area.

In the present study, the prevalence of infection by intestinal protozoa among pregnant women was 20.5% which was higher than a study done in Gondar town, Northwest Ethiopia [12]. This difference might be due to the difference in the detection method used to identify intestinal parasites. FECT which has higher sensitivity than direct microscopy was used as means of diagnosis in the present study.

Hookworm infection (18.6%) was the predominant intestinal parasitic infection among pregnant women in the present study. This finding was comparable with earlier studies in Kenya [15], East Wolega, Ethiopia [17], and Southeast Nigeria [18], but lower than a study done in Eastern Ethiopia [9] and lower than a study done Nepal [19]. The difference might be due to the difference in shoe-wearing habit and the level exposed to contaminated soil with hookworm larvae that penetrate the human skin. Working bare hands and walking barefoot are the major means of transmission for hookworm infection.

Infections with *E*. *histolytica/dipar*, *G*. *lamblia*, hookworm, and *A*. *lumbricoids* parasites are the most common infection in rural areas, and their transmission is closely associated with socioeconomic status, poor sanitation, and absence of adequate safe drinking water supplies [20].

In the present study, the prevalence of intestinal parasitic infection was higher among rural than urban dwellers. Similar findings were reported in Bahir Dar city, Northwest Ethiopia [13]; East Wolega, Ethiopia [17]; and Hossana, Eastern Ethiopia [9]. This high prevalence might be due to a lack of awareness on the transmission of intestinal parasitosis and open defecation problem in rural areas. Pregnant women always work on their field which is contaminated by night soils and eat their food with their contaminated hands. Generally, pregnant women living in rural areas had poor personal and environmental sanitation practices, low socioeconomic status, lack of awareness, and illiteracy. As a result, possibility of being infected by intestinal parasitic infection is high.

In the current study, pregnant women who were farmers, eat raw vegetables, did not utilize latrine properly, did not keep their environment clean, had the habit of soil eating, and did not get enough health education on intestinal parasitic infections had the highest probability to be infected by intestinal parasites infection. Similar findings were found in Northwest Ethiopia [13] and East Wolega, Ethiopia [17]. This might be due to a lack of awareness and the absence of education. Farmer pregnant women have limited knowledge about how and when intestinal parasites are transmitted. At the same time, regular health education is not given to pregnant women about how intestinal parasites are transmitted. As a result, eating raw vegetables, open defecation, living in unclean environment, and eating soil during pregnancy are a common phenomenon.

In the present study, pregnant women who had irrigation practice were more exposed than the one who did not have irrigation practice. This finding was comparable with previous studies in Northern Ghana [21]. This might be due to manipulating the irrigation activity barefoot and bare hands which leads to parasites like hookworm to enter by skin penetration. Moreover, water for irrigation is not clean and individuals who have the habit of eating food after cleaning their hands with such water have a posiblity to ingest the parasites.

### 4.1. Limitation of the Study

The use of only FECT for the laboratory detection of intestinal parasitic is the limitation of the study.

## 5. Conclusions

Intestinal parasitosis is a major health problem among pregnant women in the study area. Hookworm, *E*. *histolyica/dipar*, and *G*. *Lamblia* infections are the most prevalent ones. Low hygienic and sanitation habits and lack of awareness about intestinal parasitic infections were the major determinant factors for the high prevalence. Therefore, integrated health education on the means of transmission, impact, and prevention of intestinal parasitic infection should be given for pregnant women. The policymakers should also adopt a health education strategy on intestinal parasitic infection prevention through health extension workers to pregnant women. In addition, further large-scale studies should be conducted with a large sample size in the region.

## Figures and Tables

**Table 1:** Sociodemographic distribution of pregnant women with respective intestinal parasitosis in West Gojjam Zone, Northwest Ethiopia, 2017 (*N* = 578).

Variables		Positive, *N* (%)	Negative (*N*, %)	Total (*N*, %)	*x^2^*, *p* value
Age	<35 years	28 (3.8)	249 (33.5)	277 (37.3)	0.00, 0.54
	≥35 years	47 (6.3)	419 (56.4)	466 (62.7)	
Religion	Christian	276 (37.1)	460 (61.9)	736 (99)	1.60, 0.20
	Muslim	1 (0.2)	6 (0.8)	7 (1)	
Residence	Rural	187 (25.2)	276 (37.1)	463 (62.3)	5.1, 0.02
	Urban	90 (12.1)	190 (25.6)	280 (37.7)	
Woreda of West Gojam	Fenote Selam	85 (11.4)	65 (8.8)	150 (20.2)	33.34, 0.00
	Jabi Tihenan	43 (6)	102 (13.7)	151 (20.3)	
	Bure	44 (6)	98 (13.1)	142 (19.1)	
	Debube Achefer	48 (6.5)	102 (13.7)	150 (20.2)	
	Mecha	57 (7.7)	93 (12.5)	150 (20.2)	
Occupation	Employee	12 (1.6)	16 (2.2)	28 (3.8)	22.04, 0.00
	Farmer	82 (11)	88 (11.9)	170 (22.9)	
	Merchant	0	2 (0.3)	2 (0.3)	
	Labourer	5 (0.7)	1 (0.1)	6 (0.8)	
	Housewife	159 (21.4)	307 (41.3)	466 (62.7)	
Education	Illiterate	132 (17.7)	208(27.1)	340 (45.8)	10.56, 0.06
	Read & write	68 (9.2)	137 (18.4)	205 (27.6)	
	Primary	43 (5.8)	45 (6)	88 (11.8)	
	Junior	4 (0.5)	5 (0.7)	9 (1.2)	
	Secondary	26 (3.5)	55 (7.4)	81 (10.9)	
	12 complete	4 (0.5)	16 (2.2)	20 (2.7)	
Total		277 (37.3)	466 (62.7)	743	

**Table 2:** The distribution of intestinal parasite infection among pregnant women in each woredas, 2017.

Types parasitic infect	Types of parasites	West Gojjam Zone woredas					
		Burie (*N*)	Debube Achefer (*N*)	Finote Selam (*N*)	Jabi Tihnan (*N*)	Mecha (*N*)	Total (*N*)
Single infection	Hookworm	11	16	42	40	9	118
	*A*. *lubricoides*	0	1	0	1	0	2
	*S*. *mansoni*	0	0	1	0	0	1
	*E*. *vermicularis*	0	0	0	1	0	1
	*E*. *histolytica* or *E*. *dispar*	14	24	11	0	38	87
	*G. lamblia*	16	5	6	0	7	34
Double infection	HW+AL	0	0	0	1	0	1
	HW+TT	0	0	1	0	0	1
	AL+TT	0	1	0	0	0	1
	HM+EH	1	1	8	0	2	12
	HW+GL	0	0	3	0	0	3
	SS+GL	0	0	2	0	0	2
	GL+EH	1	0	9	0	1	11
Triple infection	HW+GL+EH	1	0	2	0	0	3
Total		44 (5.9)	48 (6.5)	85 (11.4)	43 (5.8)	57 (7.7)	277 (37.3)

^∗^HW: hookworm; EH: *E*. *histolytica*; GL*: G*. *lamblia*; SM: *S*. *mansoni*; AL: *A*. *lumbricoides*; SS: *S*. *stercoralis*; TT: *T*. *trichiura*; EM: *E*. *vermicularis*.

**Table 3:** Multivariable analysis showing the associated factors of intestinal parasitic infections among pregnant women in West Gojjam Zone, 2017.

Variables		Intestinal parasite		COR (95% CI)	AOR (95% CI)	*p* value
		Infected (*N*)	Noninfected (*N*)			
Address	Rural	101	362	0.33 (0.21-0.54)	2.99 (1.85-4.85)	0.00
	Urban	36	244	1	1	
Occupation	Farmer	50	87	1.90 (1.20-3.03)	1.91 (1.20-3.03)	0.01
	Non farmer	87	486	1	1	
Eating raw vegetables	Yes	131	222	6.89 (4.26-11.15)	1.45 (0.09-0.24)	0.00
	No	6	384	1	1	
Proper latrine utilization	No	20	117	0.35 (0.14-0.85)	2.89 (1.18-7.08)	0.02
	Yes	159	447	1	1	
Environmental sanitation	No	16	207	1	1	
	Yes	121	399	5.33 (2.14-13.24)	0.19 (0.08-0.47)	0.00
Soil eating habit	Yes	36	72	2.36 (1.38-4.03)	0.42 (0.25-0.72)	0.00
	No	101	534	1	1	
Irrigation practice	Yes	98	305	2.12 (1.29-3.49)	0.47 (0.29-0.77)	0.00
	No	39	301	1	1	
Health education	No	130	517	3.13 (1.30-7.58)	0.32 (0.13-0.77)	0.02
	Yes	7	89	1	1	
Environmental sanitation	No			2.29 (1.44-3.65)	1.90 (0.75-4.79)	0.07
	Yes			1	1	

## Data Availability

The whole data used to support the findings of this study have been deposited in the Hindawi repository or Freely in Google scholar: https://scholar.google.com/, Elsevier: https://www.elsevier.com, Bahir Dar University research gate: https://www.researchgate.net/institution/Bahir_Dar_University, and Research gate: https://www.researchgate.net/profile/Tadesse_Jember.
